# CD271 regulates the proliferation and motility of hypopharyngeal cancer cells

**DOI:** 10.1038/srep30707

**Published:** 2016-07-29

**Authors:** Mai Mochizuki, Keiichi Tamai, Takayuki Imai, Sayuri Sugawara, Naoko Ogama, Mao Nakamura, Kazuto Matsuura, Kazunori Yamaguchi, Kennichi Satoh, Ikuro Sato, Hozumi Motohashi, Kazuo Sugamura, Nobuyuki Tanaka

**Affiliations:** 1 Division of Cancer Stem Cell, Miyagi Cancer Center Research Institute, Natori, Japan; 2 Department of Oncovirology, Tohoku University Graduate School of Medicine, Sendai, Japan; 3 Department of Cancer Stem Cell Research, Tohoku University Graduate School of Medicine, Sendai, Japan; 4 Department of Head and Neck Surgery, Miyagi Cancer Center, Natori, Japan; 5 Division of Cancer Biology and Therapeutics, Miyagi Cancer Center Research Institute, Natori, Japan; 6 Molecular and Cellular Oncology, Miyagi Cancer Center Research Institute, Natori, Japan; 7 Department of Head and Neck Oncology, Tohoku University Graduate School of Medicine, Sendai, Japan; 8 Department of Pathology, Miyagi Cancer Center, Natori, Japan; 9 Department of Cancer Pathology, Tohoku University Graduate School of Medicine, Sendai, Japan; 10 Department of Gene Expression Regulation, Institute of Development, Aging and Cancer, Tohoku University, Sendai, Japan; 11 Department of Cancer Biology and Therapeutics, Tohoku University Graduate School of Medicine, Sendai, Japan

## Abstract

CD271 (p75 neurotrophin receptor) plays both positive and negative roles in cancer development, depending on the cell type. We previously reported that CD271 is a marker for tumor initiation and is correlated with a poor prognosis in human hypopharyngeal cancer (HPC). To clarify the role of CD271 in HPC, we established HPC cell lines and knocked down the CD271 expression using siRNA. We found that CD271-knockdown completely suppressed the cells’ tumor-forming capability both *in vivo* and *in vitro*. CD271-knockdown also induced cell-cycle arrest in G_0_ and suppressed ERK phosphorylation. While treatment with an ERK inhibitor only partially inhibited cell growth, *CDKN1C*, which is required for maintenance of quiescence, was strongly upregulated in CD271-depleted HPC cells, and the double knockdown of *CD271* and *CDKN1C* partially rescued the cells from G_0_ arrest. In addition, either CD271 depletion or the inhibition of CD271-RhoA signaling by TAT-Pep5 diminished the *in vitro* migration capability of the HPC cells. Collectively, CD271 initiates tumor formation by increasing the cell proliferation capacity through CDKN1C suppression and ERK-signaling activation, and by accelerating the migration signaling pathway in HPC.

Head and neck squamous cell carcinoma (HNSCC) is the sixth most common cancer, worldwide. HNSCC is a general classification given to various independent cancers, including those of the oral cavity, nasopharynx, oropharynx, and hypopharynx^1^. Hypopharyngeal cancer (HPC) accounts for approximately 10% of all HNSCCs. Unfortunately, approximately 80% of the HPC patients diagnosed are in the advanced stages of the disease and frequently develop delayed regional lymph node metastases or distant metastases during the course of the disease^2^. Thus, the prognosis for HPC patients remains poor, indicating the need for innovative treatment strategies.

CD271, also known as the p75 neurotrophin receptor, is a member of the tumor necrosis factor receptor (TNFR) superfamily, which binds to several ligands including nerve growth factor (NGF), brain-derived neurotrophic factor (BDNF), neurotrophine-3 (NT3), and neurotrohine-4 (NT4). Like other members of the TNFR superfamily, CD271 plays opposing roles in the development of several cancers. CD271 accelerates cell proliferation in oral cancer^3^, melanoma^4^, breast cancer^5^,^6^, brain tumors^7^, and normal myoblasts^8^. In contrast, the same receptor acts to suppress tumor growth or induce apoptosis in prostate cancer^9^, gastric cancer^10^, bladder tumors^11^, and medulloblastoma^12^. Moreover, CD271 is a negative prognostic factor in melanoma^4^, breast cancer^5^,^6^, and HPC^13^, but is a positive prognostic factor in gastric cancer^10^. In glioma cells, CD271 plays a critical role in actin fiber formation *via* a RhoA-dependent pathway^14^. These findings suggest that CD271-mediated downstream pathways vary in different cell types and tissues, and may also vary in response to different ligands. Consistent with this possibility, CD271-mediated proliferative signaling is stimulated by NGF^15^,^16^,^17^, while CD271-mediated apoptotic signaling is stimulated by proNGF^18^.

CDKN1C, a member of the Cip/Kip family (which also includes CDKN1A and CDKN1B), inhibits cyclin/CDK complexes, resulting in G_0_ phase arrest^19^,^20^. *CDKN1C* is generally known as a tumor suppressor gene, and is downregulated in many cancers^21^. Although several mechanisms for this CDKN1C inactivation have been reported, including methylation of the promoter region, histone modification, microRNA regulation, and proteasomal degradation, the precise pathway is still unknown.

We recently demonstrated that CD271 is a specific marker of HPC tumor initiation, and that it is expressed at the invasive front of the tumor^13^, suggesting that CD271^+^ cells are invasive cancer cells. However, the precise function of CD271 and the downstream signaling pathways in HPC are still obscure. Here we investigated the functional role of CD271 in tumor initiation, and in tumor cell proliferation and migration in HPC.

## Results

### CD271 is expressed in highly proliferative, undifferentiated cells in severe dysplasia and squamous cell carcinoma of the hypopharynx

**Figure 1:**
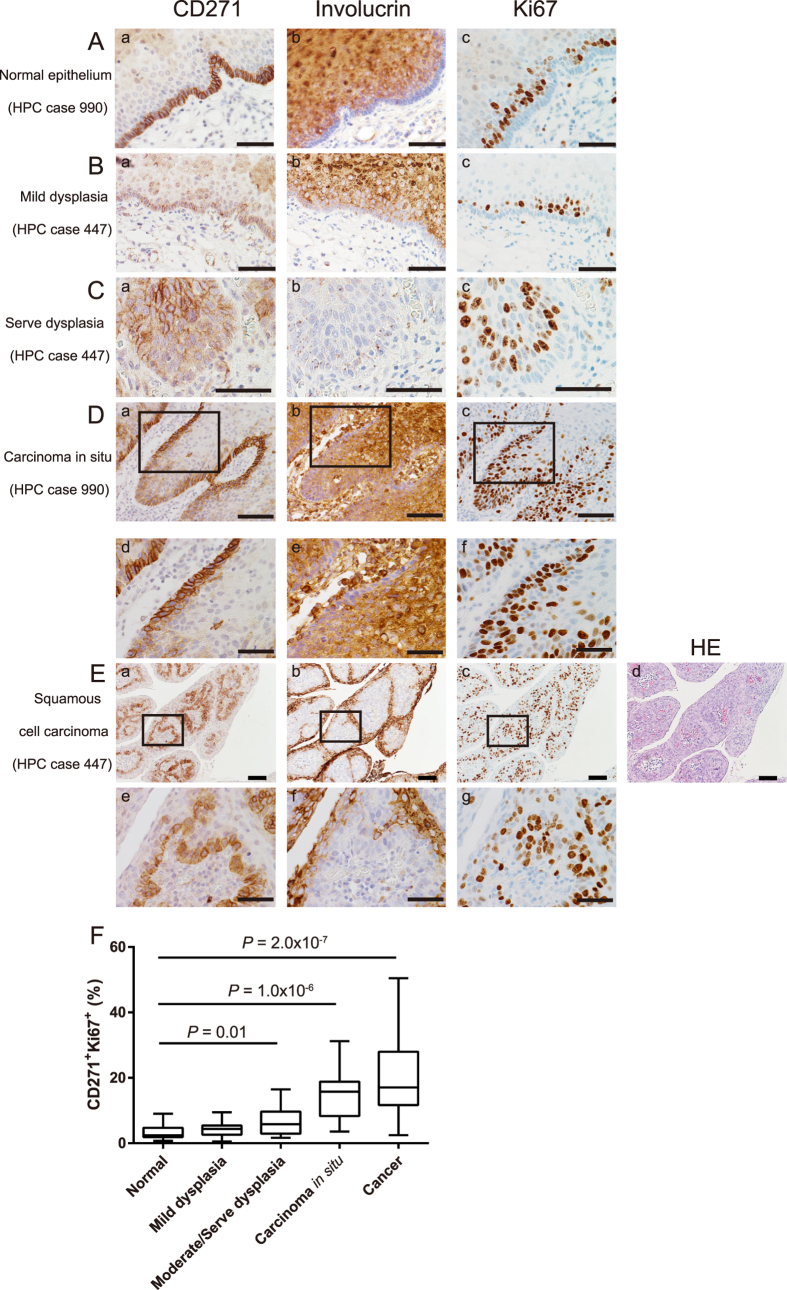
(**A**–**E**) Representative images of the immunohistochemical staining of HPC specimens obtained from 21 patients by surgical resection. Immunostaining was performed with anti-CD271 (a), anti-involucrin (b), and anti-Ki67 (c) antibodies. HE staining was also performed (**E**: d). The specimens were derived from normal epithelium (**A**: a–c), mild dysplasia (**B**: a–c), severe dysplasia (**C**: a–c), carcinoma *in situ* (**D**: a–f), and squamous cell carcinoma (**E**: a–g). (**D**: d–f) and (**E**: e–g) are high-magnification images. Bar, 50 μm (**A**: a to **C**: c, (**D**: d–f) and (**E**: e–g), 100 μm (**D**: a–c, (**E**): a–d). Note that the CD271^−^Ki67^−^ involcurin^−^ cells in the center of the nodules in (**E**) were stromal tissue (see HE staining (d)). (**F**) Quantification of the CD271^+^Ki67^+^ cells using immunofluorescently stained images and TissueFACS software (see Supplementary Figure S1 and Material and Methods). The bottom of the box is the 25th percentile and the top is the 75th. The whiskers extend to the highest and lowest observation. Number of analyzed fields: normal: *n* = 18, mild dysplasia: *n* = 22, moderate/serve dysplasia: *n* = 15, carcinoma *in situ*: *n* = 13, and cancer: *n* = 44.

To examine the role of CD271 in cell proliferation *in vivo*, we stained tissue specimens derived from human HPC patients with antibodies to CD271, Ki67, a cell proliferation marker, and involucrin, a differentiation marker. In normal epithelium, CD271^+^ cells were localized to the basal layer of the mucosa, adjacent to the basement membrane (Fig. 1A), whereas involucrin and Ki67 were undetectable in the basal layer. Similar expression patterns of the three markers were also observed in mild dysplasia (Fig. 1B). In severe dysplasia, CD271^+^Ki67^+^ cells were observed in the suprabasal layer; in the basal layer, CD271^+^ cells were present but Ki67 was undetectable (Fig. 1C). However, in carcinoma *in situ* (CIS) and squamous cell carcinoma (SCC), CD271^+^Ki67^+^ cells were observed in the basal layer, and the number of CD271^+^Ki67^−^ cells was diminished (Fig. 1D,E). In addition, the involucrin-positive cells showed a strong tendency to be CD271^−^ and *vice versa*, and this mutually exclusive expression was evident in the normal, dysplastic, and carcinoma tissues (Fig. 1A–E). Quantification of the immunofluorescence staining results revealed that the CD271^+^Ki67^+^ population was significantly increased in moderate/severe dysplasia, carcinoma *in situ*, and squamous cell carcinoma as compared with normal epithelial tissue, and that the CD271^+^Ki67^+^ population tended to increase from normal to increasingly cancerous tissue (Fig. 1F, Supplementary Figure S1). Taken together, these results suggested that CD271 expression was restricted to the dormant cells of normal epithelium and to vigorously proliferating and undifferentiated cells in severe dysplasia and in malignant tissues in the hypopharynx.

### CD271^high^ cells have highly tumorigenic and proliferative characteristics in human HPC cell lines

**Figure 2:**
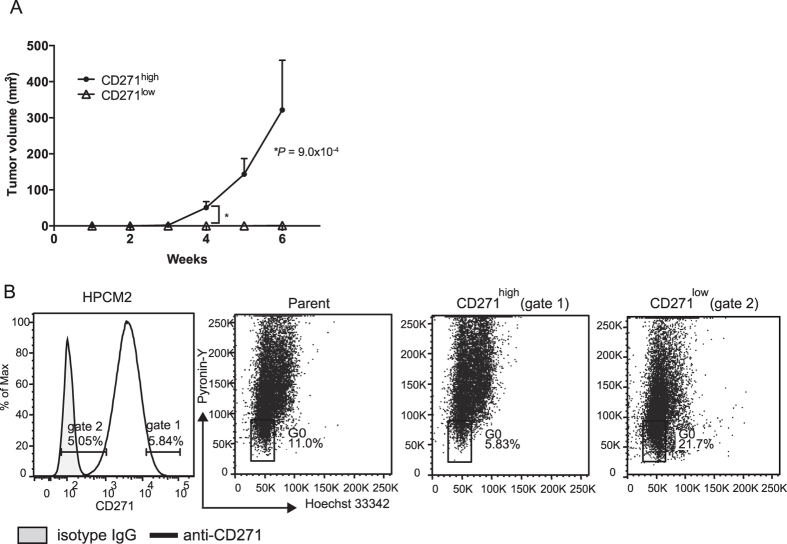
HPCM2 cell line characteristics. (**A**) HPCM2 cells were sorted into CD271^high^ and CD271^low^ cell populations by FACS and their purities were confirmed (Supplementary Figure S2). The sorted populations were inoculated into NOG mice and tumor formation was observed for six weeks. (**B**) Cell cycle analysis of the parent and sorted HPCM2 cells was performed by Hoechest 33342, pyroninY, and anti-CD271 antibody staining (see Materials and Methods). Gated fractions of the CD271^high^ population (gate 1, 5.84% of the cells) and CD271^low^ population (gate 2, 5.05% of the cells) and the entire population of the parent cells were subjected to cell cycle analysis. Quantification of the G_0_ populations is shown.

We previously established HPC patient-derived xenograft (PDX) lines by transferring tumor specimens into immunodeficient NOD/scid/γcnull (NOG) mice^13^. In the present study, we analyzed two established cell lines, HPCM2 and HPCM7, which were derived from squamous cell carcinoma-derived PDX lines. First, we examined the tumorigenicities of CD271^high^ and CD271^low^ cells sorted from the HPCM2 and HPCM7 cells lines. The purity of the CD271^high^ and CD271^low^ cell populations was confirmed by flow cytometry (Supplementary Figure S2), and then the cells were injected into NOG mice to determine their tumorigenicities. After six weeks of engraftment, we found that the HPCM2 CD271^high^ population had formed tumors at all four (4/4) injection sites, while the CD271^low^ population had formed a tumor in only one out of four (1/4) injection sites (Fig. 2A, Table 1A). Similarly, the HPCM7 CD271^high^ population formed tumors at all four injection sites, while the corresponding CD271^low^ cell population did not form tumors at any of the injection sites (Supplementary Figure S3A, Table 1B). These results were consistent with our previously reported findings^13^. Next, to determine the *in vitro* proliferative capability of the CD271^high^ and CD271^low^ populations, cultured HPCM2 and HPCM7 cells were subjected to flow cytometry analysis (Fig. 2B, Supplementary Figure S3B). The CD271^high^ populations of both cell lines were found to be primarily cycling through the S and G_2_/M phases, while the CD271^low^ populations were primarily in the G_0_ phase. These findings were consistent with the staining patterns of CD271 and Ki67 in the HPC tissue (Fig. 1D–F), and indicated that CD271^high^ HPC cells were highly proliferative both *in vitro* and *in vivo*.

### CD271 knockdown inhibits cell cycle progression

**Figure 3:**
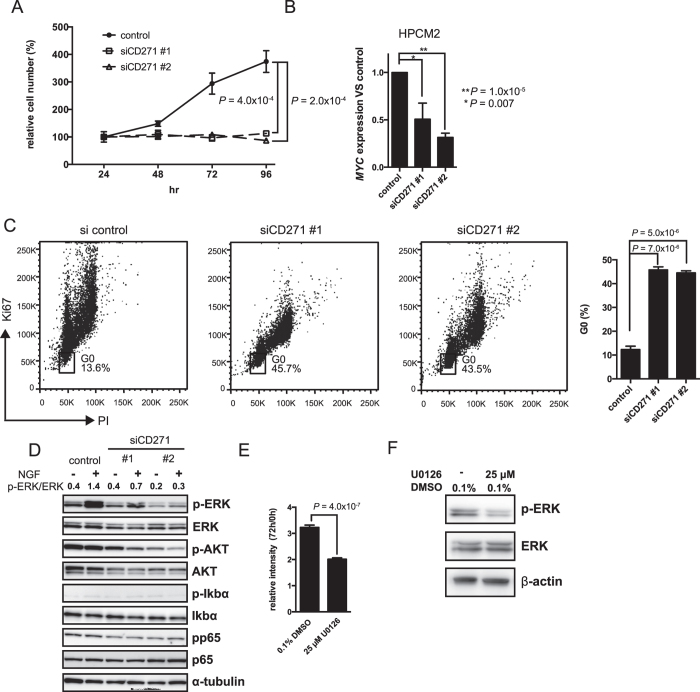
CD271-knockdown in HPCM2 cells reduces *in vitro* proliferation. (**A**) Proliferation of CD271-knockdown (siCD271#1 and siCD271#2) and control HPCM2 cells was assessed with MTT assays. (*n* = 3) (**B**) HPCM2 cells were transfected with the siRNAs, incubated for 48 h, and then *MYC* expression profiles in CD271-knockdown and control HPCM2 cells were determined by real-time PCR. (*n* = 3) (**C**) Cell cycle analysis of CD271-knockdown cells was performed by Ki67 and PI staining. The percentage of cells in G_0_ is shown in the graph (*n* = 3). (**D**) Western blot analysis of ERK, AKT, Iκbα, and p65 and their phosphorylated forms (p-ERK (Thr202/Tyr204), p-AKT (Ser473), p-Iκbα (Ser32), and pp65 (Ser536)) was performed with HPCM2 cells incubated for 24 h in serum-free medium and then treated with 100 ng/ml NGF for 10 min. p-ERK/ERK, the ratio of p-ERK to total ERK based on band density analysis with ImageJ software. (**E**) HPCM2 proliferation assays after treatment with U0126. The relative intensity values represent the ratio of the MTT absorbance after the cells were incubated for 72 h with U0126 to that measured at 0 h of treatment. (*n* = 4) (**F**) ERK phosphorylation was analyzed by Western blotting after 72 hours of U0126 treatment. PI, propidium iodide.

To assess the functional involvement of CD271 in cell cycle progression, we performed CD271-knockdown in HPCM2 and HPCM7 cells by introducing small interfering RNAs (siRNAs). Two CD271-targeting siRNAs, siCD271 #1 and #2, were transfected into the cell lines, and their ability to suppress CD271 expression was confirmed by flow cytometry (Supplementary Figure S4A,B). We then examined the effect of both siRNAs on *in vitro* cell proliferation, the expression of *MYC*, a key regulator of cell proliferation in cancer, and cell cycling. Cell viability analysis using MTT assays showed that CD271-knockdown in HPCM2 resulted in the complete loss of cell proliferation (Fig. 3A, dashed lines). CD271-knockdown also decreased the cell proliferation in another HPC cell line, HPCM7 (Supplementary Figure S5A). Real-time PCR analysis revealed that the *MYC* expression was significantly decreased in the CD271-knockdown cells (Fig. 3B, Supplementary Figure S5B), and cell cycle analysis showed that CD271-knockdown caused the cells to undergo cell cycle arrest in G_0_ (Fig. 3C, Supplementary Figure S5C). These results indicated that CD271 played a crucial role in cell cycle progression in HPC cells.

Since CD271 promotes cell proliferation through the activation of AKT and mitogen-activated protein kinase (MAPK)/ERK signaling^22^, we investigated the effect of CD271 knockdown on this proliferative signaling in HPCM2 cells. The cells were transfected with the CD271 siRNAs and assayed for the nerve growth factor (NGF)-induced phosphorylation of ERK, AKT, p65, and IκBα. NGF stimulation led to increased levels of phosphorylated ERK (p-ERK), which were reduced by CD271-knockdown. In contrast, the phosphorylation of AKT, p65, and IκBα was unaffected by NGF stimulation, although the AKT and phosphorylated AKT (p-AKT) levels were reduced by CD271 knockdown, irrespective of NGF stimulation (Fig. 3D). We next analyzed the effect of a MEK-ERK inhibitor, U0126, on proliferation (Fig. 3E) and ERK phosphorylation in HPCM2 cells (Fig. 3F). Both cell proliferation and ERK phosphorylation were clearly reduced by U0126 treatment.

### CDKN1C plays a critical role in CD271-knockdown-dependent cell cycle arrest

**Figure 4:**
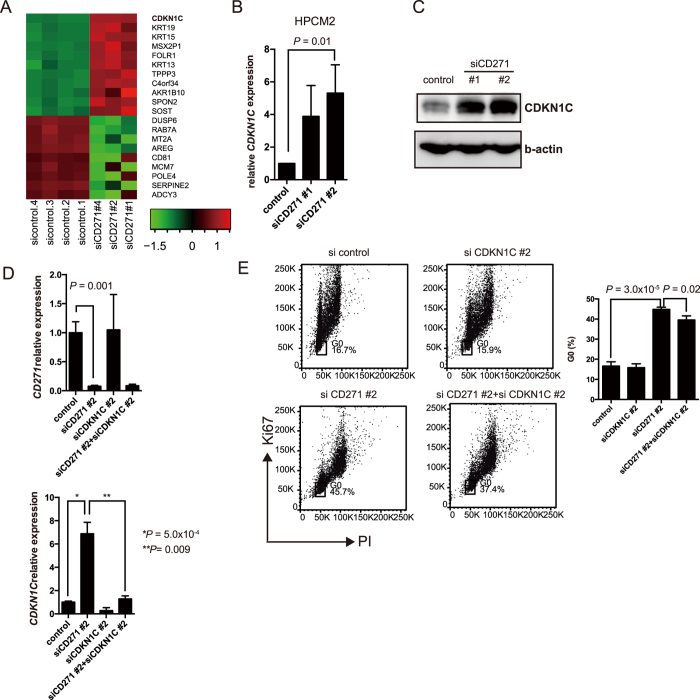
CD271-knockdown cells exhibit altered expression of a cell cycle-related gene. (**A**) Heatmap showing the expression profile of 20 genes in CD271-knockdown (siCD271 #1, 2, and 4) and control (sicontrol. 1–4) cells as determined by microarray analysis using the weighted average difference (WAD) algorithm^23^. Red colors indicate higher abundance, and green colors indicate lower abundance (Z-score). (**B**) The expression profile of *CDKN1C* in CD271-knockdown and control HPCM2 cells was analyzed using real-time PCR. (*n* = 3) (**C**) Expression levels of CDKN1C in CD271-knockdown and control cells were analyzed by Western blotting. (**D**) Expression profiles of *CD271* (upper) and *CDKN1C* (lower) in control, CD271-knockdown, CDKN1C-knockdown, and CD271/CDKN1C double-knockdown HPCM2 cells were analyzed by real-time PCR. (*n* = 3) (**E**) Cell cycle analysis of CD271- and/or CDKN1C-knockdown HPCM2 cells. The percentages of cells in G_0_ in the cell samples are shown in the graph (*n* = 3). PI, propidium iodide.

Next, we compared gene expression profiles between the CD271 knockdown and control HPCM2 cells by microarray analysis using a weighted average difference (WAD) algorithm^23^ (Fig. 4A). The datasets were also analyzed with gene set enrichment analysis (GSEA)^24^ to identify differentially expressed gene sets in the knockdown versus control cells (Supplementary Table S1). Among the top 29 Gene Ontology gene sets identified with a false discovery rate (FDR) *q*-value < 0.250, 14 cell cycle-related pathways (Supplementary Table S1, shown in red) were found to be differentially expressed in the CD271-knockdown versus control HPCM2 cells.

Analysis of the microarray data showed that *CDKN1C*, a cell cycle-related gene, was differentially expressed in the control and knockdown HPCM2 cells (Fig. 4A). We next examined the gene expression profiles of *CDKN1A*, *CDKN1B*, and *CDKN1C* in the CD271-knockdown versus control cells, using real-time PCR. We found marginal differences in the *CDKN1A* and *CDKN1B* expression level between the CD271-knockdown and control HPCM2 cells (Supplementary Figure S6), whereas the *CDKN1C* expression was significantly higher in the knockdown cells than in the control cells (Fig. 4B). A similar result was obtained in HPCM7 cells (Supplementary Figure S7). We also detected an increased expression of CDKN1C protein in the CD271-knockdown cells (Fig. 4C).

On the basis of these observations, we hypothesized that the CD271 knockdown-induced G_0_ arrest was mediated by the increased expression of *CDKN1C*. To test this hypothesis, we transfected HPCM2 cells with both CD271 and CDKN1C siRNAs. The relative expression levels of both genes are shown in Fig. 4D. Cell cycle analysis showed that the CD271-knockdown cells were primarily in G_0_ phase and that the G_0_ arrest was partially rescued by the CD271-CDKN1C double knockdown (Fig. 4E). These results suggested that CD271 controls the cell cycle in part by regulating CDKN1C expression.

### CD271 is required for HPCM2-mediated tumorigenicity

**Figure 5:**
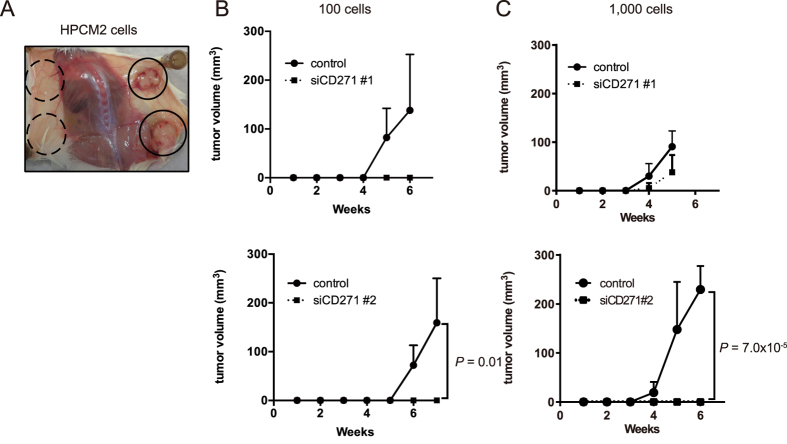
Tumorigenicity of CD271-knockdown and control HPCM2 cells. (**A**) A representative autopsy of a NOG mouse injected with CD271-knockdown (siRNA #2) HPCM2 cells (dotted circles) and control cells (solid circles), 100 cells. (**B,C**) Tumor-initiating activities of HPCM2 cells transfected with siCD271 #1 (upper) and siCD271 #2 (lower). (**B**) 100 cells, (**C**) 1,000 cells. (*n* = 4).

To determine the effect of CD271 on HPCM2 cell-mediated tumorigenicity, the two types of CD271-knockdown (siCD271 #1 and #2) HPCM2 cells and control HPCM2 cells were xenografted into NOG mice. When 1,000 cells were injected, the control cells formed tumors at seven out of eight sites (7/8), whereas the CD271-knockdown cells (#1) formed tumors at three out of four sites (3/4) and the CD271-knockdown cells (#2) formed no tumor at any of four sites, 5 weeks after the xenograft. When 100 cells were injected, a clear difference between the CD271-knockdown and control cells was apparent six weeks after the xenograft (control: 7/8, siCD271 #1: 0/4, siCD271 #2: 0/4) (Fig. 5, Table 2). These results suggested that the *in vivo* proliferation of HPCM2 cells was primarily mediated by CD271.

### Involvement of CD271 in cell migration

**Figure 6:**
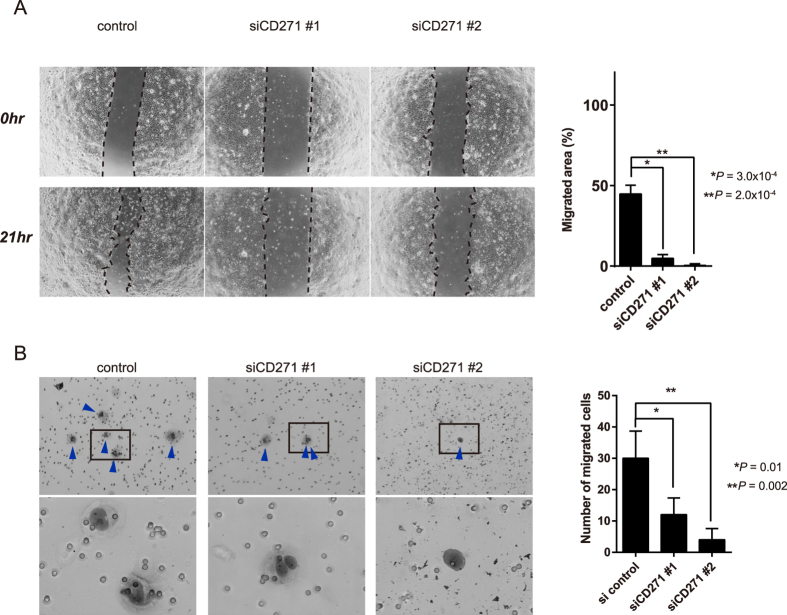
CD271-knockdown reduces the *in vitro* migration of HPCM2 cells. (**A**) Scratch assays. Confluent HPCM2 cells were scratched with a pipette just after transfection with siCD271#1 or #2, and the scratched areas were measured at 0 and 21 h of culture. Left: The migrated areas are indicated by dotted lines. Right: Quantification of the migrated areas. (*n* = 6). (**B**) Left: Representative images of the migrated cells in a transwell migration assay. The blue arrowheads in the top images indicate migrated cells, and the bottom images are high-magnification pictures. CD271-knockdown and control HPCM2 cells were incubated in a transwell unit for 72 h in medium containing 10% FBS, and then the transwell membrane was stained with Diff-Quick. Right: Quantification of the migrated cells. (*n* = 4).

**Figure 7:**
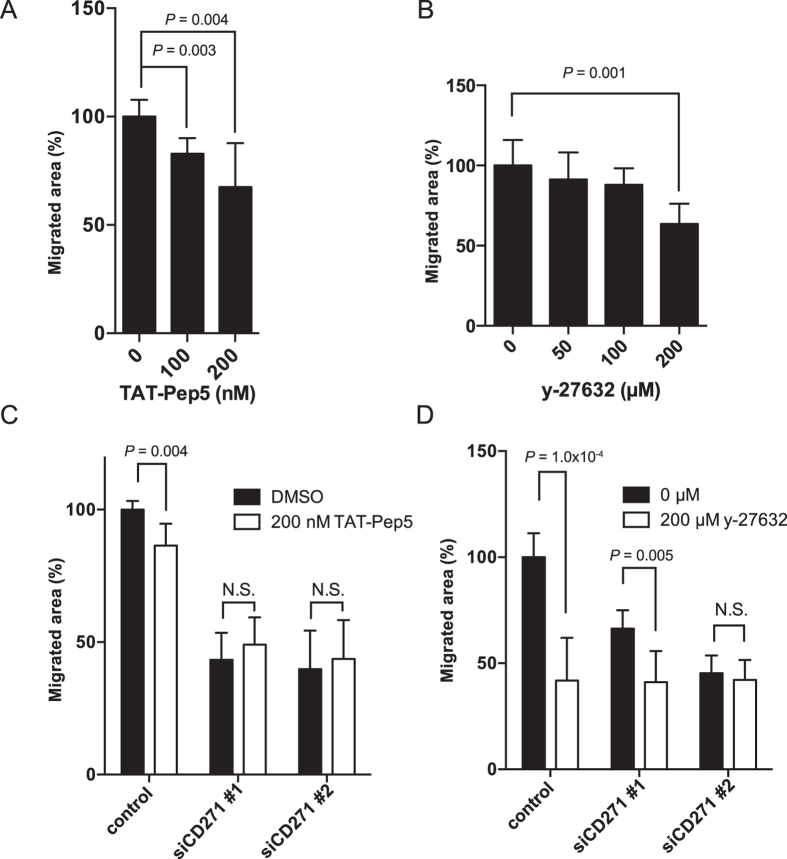
RhoA and ROCK inhibitors inhibit CD271-mediated HPCM2 cell migration. Effects of a CD271-specific inhibitor, TAT-Pep5 (**A,C**) and a ROCK inhibitor, Y-27632 (**B,D**) on the migration activity of HPCM2 cells (**A,B**) and siCD271-transfected HPCM2 cells (**C,D**). HPCM2 cells were transfected with the siRNAs, incubated for 24 h, and then treated with 200 nM TAT-Pep5 or 200 μM Y-27632, and incubated for another 24 h before scratching. (*n* = 6).

Since we previously reported that CD271^high^ cells are localized to the invasive front of HPC tumours^13^, we speculated that CD271 may be involved in tumor cell migration. To investigate this possibility, we performed scratch assays with CD271-knockdown and control HPCM2 cells, and added 5 μg/ml mitomycin-C to the culture three hours before the scratch to avoid the effect of cell proliferation. The CD271-knockdown cells exhibited significantly reduced cell motility compared to the control cells (Fig. 6A). Similar results were obtained when the knockdown and control cells were analyzed in transwell migration assays (Fig. 6B), which were also performed with mitomycin-C-treated cells to avoid cell-proliferation effects. CD271-knockdown HPCM7 cells also showed reduced cell motility (Supplementary Figure S8A,B). Since CD271 regulates RhoA activation and cell migration in gliomas^14^, we tested the effects of TAT-Pep5, a CD271-RhoA inhibitor, and Y-27632, a Rho-associated, coiled-coil containing protein kinase (ROCK) inhibitor, on HPCM2 migration. HPCM2 cells treated with TAT-Pep5 or Y-27632 showed decreased migration capabilities compared to untreated cells (Fig. 7A,B). The difference of the migration capability was smaller between CD271-knockdown and control cells in the absence and presence of the inhibitors (Fig. 7C,D). These results indicated that CD271 regulated cell migration *via* the RhoA-ROCK pathway in HPC cells.

## Discussion

Here, we undertook a number of *in vitro* and *in vivo* studies to investigate the role of CD271 in tumor development in HPC. We found that CD271 and Ki67 were colocalized in squamous cell carcinoma but not in the normal epithelium or mild dysplasia of HPC specimens, suggesting that CD271^+^ cells were highly proliferative in tumors, but dormant under normal conditions. Similar observations were also reported in esophageal cancer^25^. Using two *in vitro* established cell lines, HPCM2 and HPCM7, which were derived from HPC tumor xenografts, we found that the CD271^+^ cell population was highly proliferative, and that the knockdown of CD271 induced cell cycle arrest in G_0_. These data indicate that CD271 plays a critical role in HPC cell proliferation by regulating the cell cycle. Such contribution of CD271 to cell proliferation has been reported in several cancers including oral cancer^3^, melanoma^4^, breast cancer^5^,^6^ and brain tumors^7^. In contrast, however, CD271 is also known to induce cell apoptosis through G_0_/G_1_ cell cycle arrest in prostate cancer^26^ and gastric cancer^10^. Furthermore, the knockdown of CD271 has been demonstrated to induce cell cycle arrest in G_2_/M in oral cancer^3^. These various signaling pathways are thought to have relevance to multiple ligand-CD271 interactions^22^. For example, NGF binds CD271 and activates the ERK^15^, AKT^16^, and NF-κB^17^ pathways, leading to cell proliferation, whereas the proNGF binding of CD271 activates PTEN^27^ and caspases 3, 6, and 9^28^, leading to cell cycle arrest and apoptosis. These observations suggest that CD271-mediated downstream pathways vary in different cell types and tissues, and also in response to different ligands, although we have not yet identified any CD271 ligand contributing to HPC proliferation.

We used several approaches to investigate CD271’s downstream signaling in HPCM2 cells. Since the NGF-CD271 interaction stimulates ERK, AKT, and NF-κB phosphorylation^15^,^16^,^17^, we investigated the phosphorylation of these signaling molecules in HPCM2 cells. We found that NGF enhanced the ERK phosphorylation, but not that of AKT or NF-κB in these cells, suggesting that MEK/ERK signaling may be involved in HPCM2 proliferation. Moreover, CD271-knockdown in HPCM2 cells resulted in reduced NGF-stimulated ERK phosphorylation. Although treating HPCM2 cells with a MEK/ERK inhibitor reduced HPCM2 cell proliferation, the inhibition was not complete (Fig. 3E), despite the strong reduction in ERK phosphorylation (Fig. 3F), suggesting that CD271-induced proliferation may involve additional signaling pathways. We also found that CD271-knockdown in HPCM2 cells induced cell cycle arrest in G_0_ and the upregulation of *CDKN1C*, a cell cycle inhibitor that causes arrest in G_0_. Because the induction level of *CDKN1C* was lower in MEK/ERK inhibitor-treated cells than in the CD271-knockdown cells (Supplementary Figure S9, compare with Fig. 4B), we deduced that *CDKN1C* was controlled by other CD271-related pathway(s) in addition to the MEK/ERK pathway. Collectively, our results suggest that CD271 regulates HPCM2 cell proliferation by activating the MEK/ERK pathway and by suppressing the expression of the cell cycle inhibitor, *CDKN1C*.

CD271 plays a critical role in glioma cell invasion^14^, a process that is dependent on the activation of RhoA, a key regulator of cell migration^29^. Using a scratch and migration assay, we also demonstrated that either CD271-knockdown or cell treatment with TAT-pep5, a CD271-binding RhoA inhibitor, resulted in reduced HPCM2 migration. However, the treatment of CD271-depleted cells with TAT-pep5 did not further reduce the migration of these cells. These findings suggested that CD271-mediated RhoA activation was required for tumor cell migration in HPC. Furthermore, we previously reported that CD271^+^, but not CD271^−^, cancer cells are clustered and highly expressed at the invasive front of HPC tumors^13^. Consistent with this finding, our present study revealed that CD271^+^Ki67^+^ cells were abundant at the invasive fronts of HPC surgical specimens. Taken together, our findings indicate that CD271 may play critical roles in HPC cell invasion *via* a RhoA-dependent pathway.

Thus, we conclude that CD271 may promote HPC cell proliferation and invasion, and may represent a molecular target for HPC.

## Materials and Methods

### Ethics statements

This study was conducted according to the principles expressed in the Declaration of Helsinki and was approved by the Ethics Committees at the Miyagi Cancer Center Research Institute (Natori, Japan). The animal experimental protocols were approved by the Miyagi Cancer Center Animal Care and Use Committee.

### Cell lines

The HPCM2 cell line was previously established by serial xenografts of cancer tissues derived from two human HPC patients^13^. The HPCM7 cell line was newly established from squamous cell carcinoma of HPC. The cell lines were maintained in RPMI-1640 medium supplemented with 10% fetal bovine serum (FBS) and 1% penicillin-streptomycin. The cells were passaged at least 10 times *in vitro* before being used in experiments.

### Tissue specimens

Tissue specimens were obtained from 27 patients with HPC who underwent surgical resection from 2007 to 2016 at the Miyagi Cancer Center. All of the patients provided written informed consent. Six samples were excluded from the analysis because no CD271 staining was observed in them. Histologic grades were determined according to the World Health Organization Classification of Tumors (2005).

### Antibodies

An anti-human APC-CD271 antibody (clone ME20.4-1.H4, Miltenyi Biotec, Germany) was used for flow cytometry analysis. The following antibodies were used for Western blotting analysis: anti-phospho-p44/42 MAPK (ERK1/2, Thr202/Tyr204) (20G11, Cell Signaling Technologies, Danvers, MA, USA), anti-ERK (137E5, Cell Signaling), anti-phospho-NF-κB p65 (S536) and anti-NF-κB p65 (93H1 and D14E12, Cell Signaling), anti-phospho-Iκbα (Ser32) and anti-Iκbα (14D4 and L35A5, Cell Signaling), anti-phospho- Akt (S473) and anti-Akt (pan, Cell Signaling) anti-α-tubulin (B-5-1-2, Santa Cruz Biotechnology, Dallas, TX, USA), anti-involucrin (clone SY-5, Sigma-Aldrich, St. Louis, IL, USA), anti-CDKN1C (NBP1-89917, Novus Biologicals, Littleton, CO, USA), and anti-β-actin (AC-15, Sigma-Aldrich). The following antibodies were used for immunohistochemistry analysis: anti-CD271 (C40-1457, BD Biosciences, Franklin Lakes, NJ, USA), anti-Ki67 [clone 30-9 (Roche, Switzerland) for DAB staining and clone SP6 (Abcam, UK) for immunofluorescence staining] and anti-involucrin (clone SY-5, Sigma-Aldrich).

### Small interfering RNAs

CD271 siRNAs #1 (HSS107179), #2 (HSS107181), and #3 (HSS107181), CDKN1C siRNA (HSS141530), and non-silencing control siRNA (12935-300) were purchased from Invitrogen (Carlsbad, CA, USA). The siRNA transfections were performed using Lipofectamine® RNAiMAX Reagent (Life Technologies, CA, USA) in antibiotic-free medium for 48 h. siRNA knockdown efficiencies were confirmed by flow cytometry analysis or real-time PCR.

### Immunohistostaining

Paraffin-embedded, formalin-fixed, 3-μm tissue sections from human HPC patients were deparaffinized in xylene, and rehydrated by washing with a series of ethanol dilutions and distilled water. Heat-induced epitope retrieval was performed by microwaving the sections in a pH 9.0 target retrieval solution (Dako, Denmark) for CD271 staining or 10 mM citric acid buffer (LSI Medience Corporation, Japan) for involucrin staining. Endogenous peroxidases were blocked with 0.3% H_2_O_2_. The sections were incubated with anti-human CD271 (1:4,000) for 20 min, or with anti-involucrin (1:1,000) for 90 min, at 37 °C. The CD271-stained sections were incubated for 15 min with mouse LINKER (Dako), followed by incubation with secondary antibody, and development with 3,3′-diaminobenzidine (DAB) Chromogen (EnVision™ Detection SystemsPeroxidase/DAB, Rabbit/Mouse, Dako). Immunostaining with anti-CD271 and anti-involucrin was performed according to the manufacturers’ protocols. Anti-Ki67 staining was performed on a Ventana Discovery automation system (Roche, Switzerland).

### Immunofluorescence staining and quantification of CD271 and Ki67 localization

The paraffin-embedded, formalin-fixed, 3-μm tissue sections from human HPC patients were deparaffinized, and then epitope retrieval was performed as described above. The sections were incubated with anti-human CD271 (1:4,000) and anti-Ki67 (1:100) primary antibodies for 1 h at 37 °C. The sections were then incubated for 1 h with Alexa Fluor 594 goat anti-rabbit IgG (1:200, Invitrogen), goat anti-mouse IgG (H + L) HRP conjugate (1:200, Invitrogen) and DAPI solution (1:1,000, Dojindo, Japan), and the CD271 signals were amplified using a Tyramide Signal Amplification Kit (ThermoFisher, MA, USA), according to the manufacturer’s protocol. Images were randomly obtained with a fluorescence microscope (BZ-9000, Keyence, Japan), at least three fields per specimen. Each picture was graded as normal, mild dysplasia, moderate/serve dysplasia, carcinoma *in situ*, or squamous cell carcinoma according to the World Health Organization Classification of Tumors (2005). The CD271, Ki67, and DAPI-positive cell counts were each evaluated using TissueQuest® analysis software (TissueGnostics, Austria). The percentages of CD271- and/or Ki67-positive cells were calculated by dividing the number of CD271- and/or Ki67-positive cells by the number of DAPI-stained cells in each field.

### ERK inhibition

The MEK/ERK inhibitor U0126 was purchased from InvivoGen (San Diego, CA, USA) and solubilized in DMSO. The inhibitor solution or DMSO was added to cells at the indicated concentration, and the cells were incubated in complete medium for 72 h.

### *In vitro* scratch assays

Cells were cultured on a 24-well tissue culture plate (BD Biosciences) until confluent and then treated with 5 μg/ml mitomycin C (Wako, Japan) for 3 h. The cells were then gently scratched with a pipette tip, washed with medium, and incubated with fresh medium for 21 h.

### Cell migration assays

Cell migration assays were conducted as described previously^30^ with minor modifications. After incubation for the indicated times, the membrane-adherent cells were stained with Diff-Quick (Sysmex Corporation, Japan), and the cells were counted. For the assays using CD271-knockdown cells, the cells were pre-treated with 5 μg/ml mitomycin C (Wako, Japan) for 3 h. The experiment was performed at least three times.

### Quantitative real-time PCR

Total RNA was extracted from hypopharyngeal cancer (HPCM2, HPCM7) cells using the RNeasy Mini Kit (Qiagen, Valencia, CA) or Sepasol-RNA I Super G (Nakalai Tesque, Japan), and reverse transcribed using a PrimeScript II cDNA Synthesis Kit (Takara Bio, Japan). Real-time PCR was performed using the Brilliant III Ultra- Fast SYBR Green QPCR Master Mix (Agilent Technologies). ß-actin was used as an endogenous reference gene. The primer sequences used for real-time PCR are listed in Supplementary Table S2.

### *In vivo* tumorigenesis

The tumor formation assay was performed as described previously with minor modifications^13^. For the xenotransplantation of CD271^high^ and CD271^low^ cells, live populations of HPCM2 and HPCM7 cells stained with an anti-CD271 antibody were first sorted with a FACSAria II (BD Biosciences). 7-Amino Actinomycin D (7-AAD, Sigma) was used to exclude dead cells. For the xenotransplantation of CD271-knockdown cells using siRNA^31^, HPCM2 cells were transiently transfected with siCD271#1, siCD271#2, or sicontrol. Forty-eight hours after transfection, the cells were trypsinized, and live populations were stained and sorted as described above. The cells were suspended in 50 μl of RPMI supplemented with 10% FBS and an equal volume of Matrigel matrix (BD Biosciences) at 4 °C, and then injected into NOG mice with a 1 ml syringe. Each mouse received an injection of CD271^+^ or control cells in the right side, and CD271^−^ or RNAi-treated cells in the left side. Tumor formation was monitored weekly.

### Gene expression profiling

Whole genome expression profiling of CD271-knockdown and control cells was performed with at least three biological replicates. RNA from HPCM2 cells was purified using RNeasy Mini Kits and QIAshredder columns (Qiagen, Canada). Microarray analysis was performed using SurePrint G3 Human Gene Expression 8 × 60 K Microarray Kits (Agilent Technologies, CA) and the Low Input Quick Amp Labeling Kit (Agilent Technologies) according to the manufacturer’s protocols. After hybridization and washing, the processed slides were scanned with the Agilent Microarray Scanner (Agilent Technologies), and the acquired array images were analyzed using Agilent Feature Extraction Software (Agilent Technologies). Normalization and subsequent data processing were performed using R statistical software.

### Gene-set enrichment analysis (GSEA)

Quantile normalized expression data of CD271-knockdown and control cells were analyzed with GSEA (Broad Institute, http://www.broadinstitute.org/gsea/index.jsp) and the Gene Ontology gene sets.

### Flow cytometry analysis

The anti-human APC-CD271 antibody (clone ME20.4-1.H4, Miltenyi Biotec) was used for flow cytometry analysis. The cells were incubated with the APC-CD271 antibody or control mouse IgGκ_1_ for 30 min at 4 °C, washed twice, and subsequently analyzed using a FACSCanto II (Becton Dickinson, CA).

### Cell cycle analysis

CD271-knockdown cells were fixed with 70% ethanol at −20 °C. The fixed cells were stained with anti-Ki67 (1:60)(Biolegend) and 10 μg/ml propidium iodide. To perform cell cycle analysis for live cells, the cells were incubated with 5 μg/ml Hoechest 33342 and 0.2 μg/ml pyroninY (Sigma) for 1 h at 37 °C. The stained cells were analyzed by flow cytometry.

### Western blotting

Cells (5 × 10^6^) were washed once with PBS (without calcium) and sonicated in cold buffer (20 mM Tris, 2.5 mM Sodium Pyrophosphate, 150 mM NaCl, 1 mM Disodium β-Glycerophosphate Pentahydrate, 1 mM ethylenediaminetetraacetic acid, 1 mM 14-bis[(acetyloxy)methyl] ester, 1% Triton-X) for 5 min. The lysates were clarified by brief centrifugation (10,000 × *g*), and the supernatant was used for analysis. After measuring protein concentrations with a protein assay kit (Bio Rad, Richmond, CA), 20 μg protein of each sample was mixed with an equal volume of SDS-loading buffer (100 mM Tris-Cl pH 6.8, 4% sodium dodecyl sulfate, 0.2% bromophenol blue, 20% glycerol, 2% β-mercaptoethanol), boiled for 5 min, and subjected to SDS-PAGE (Wako, Japan). The separated proteins were transferred onto a PVDF membrane (Millipore, Billerica, MA) that was incubated with 1:1,000-diluted primary antibody and then with HRP-conjugated anti-mouse or anti-rabbit antibody (Cell Signaling Technology) as recommended by the manufacturers. Primary antibody binding was detected using a West Pico chemiluminescent substrate kit (Pierce, Rockford, IL), and images were captured by a CCD camera (Fuji Film, Tokyo, Japan).

### MTT assay

A total of 5,000 HPCM2 and 10,000 HPCM7 cells were plated in 0.1 ml RPMI-1640 medium supplemented with 10% FBS and 1% penicillin-streptomycin in a 96-well plate. At the indicated times, MTT assay reagent (Roche) was added to each well according to the manufacturer’s protocol. The absorbances at 575 nm and 650 nm (background measurement) were determined using a plate reader (VersaMax ELISA Microplate Reader, Molecular Devices, Sunnyvale, CA, USA). At least three replicate wells were assayed for each condition, and the S.D. was determined.

### Statistical analysis

Statistical analysis was performed using GraphPad Prism vision 6.0b (GraphPad Software, La Jolla, CA, USA). The differences between two groups were analyzed by the unpaired *t*-test. *p*-values < 0.05 were considered to be statistically significant.

## Additional Information

**How to cite this article**: Mochizuki, M. *et al*. CD271 regulates the proliferation and motility of hypopharyngeal cancer cells. *Sci. Rep.* **6**, 30707; doi: 10.1038/srep30707 (2016).

## Supplementary Material

Supplementary Information

## Figures and Tables

**Table 1:** Tumor initiation capability of the CD271^low^ and CD271^high^ cells in HPCM2 (A) and HPCM7 (B) cells.

Population	Number of cells injected	Weeks						
(A)								
		1	2	3	4	5	6	
CD271^high^	1,000	0/4	0/4	3/4	4/4	4/4	4/4	
CD271^low^	1,000	0/4	0/4	0/4	0/4	0/4	1/4	
(B)								
		1	2	3	4	5	6	7
CD271^high^	100	0/4	0/4	3/4	4/4	4/4	4/4	4/4
CD271^low^	100	0/4	0/4	0/4	0/4	0/4	0/4	0/4

**Table 2:** (A,B) Tumor initiation capability of control versus (A) siCD271 #1 and (B) siCD271 #2 in HPCM2 cells.

Population	Number of cells injected	Weeks						
(A)								
		1	2	3	4	5	6	
Control	1,000	0/4	0/4	2/4	4/4	4/4		
siCD271 #1	1,000	0/4	0/4	0/4	1/4	3/4		
Control	100	0/4	0/4	0/4	0/4	3/4	3/4	
siCD271 #1	100	0/4	0/4	0/4	0/4	0/4	0/4	
(B)								
		1	2	3	4	5	6	7
Control	1,000	0/4	0/4	0/4	3/4	4/4	4/4	
siCD271 #2	1,000	0/4	0/4	0/4	0/4	0/4	0/4	
Control	100	0/4	0/4	0/4	0/4	1/4	4/4	4/4
siCD271 #2	100	0/4	0/4	0/4	0/4	0/4	0/4	0/4
