# Supplementary material for: CD271 regulates the proliferation and motility of hypopharyngeal cancer cells
Source: Sci Rep. 2016 Jul 29;6:30707. doi: 10.1038/srep30707 (PMC4965829; doi:10.1038/srep30707)
Supplement: Supplementary Information [file srep30707-s1.pdf]

<Title>

CD271 regulates the proliferation and motility of hypopharyngeal cancer cells

Mai Mochizuki[1,7], Keiichi Tamai[1,8], Takayuki Imai[4], Sayuri Sugawara[2], Naoko Ogama[2], Mao Nakamura[3], Kazuto Matsuura[4,9], Kazunori Yamaguchi[3,7], Kennichi Satoh[1,8], Ikuro Sato[5,10], Hozumi Motohashi[6], Kazuo Sugamura[3], Nobuyuki Tanaka[2,11]

[1]Division of Cancer Stem Cell, [2]Cancer Biology and Therapeutics, [3]Molecular and Cellular Oncology, Miyagi Cancer Center Research Institute, Natori, Japan; Department of [4]Head and Neck Surgery, [5]Pathology, Miyagi Cancer Center, Natori, Japan; [6]Department of Gene Expression Regulation, IDAC, Tohoku University, Sendai, Japan; [7]Department of Oncovirology, [8]Cancer stem cell research, [9]Head and Neck Oncology, [10]Cancer Pathology, [11]Cancer Biology and Therapeutics, Tohoku University Graduate School of Medicine, Sendai, Japan

Running Title: Role of CD271 in hypopharyngeal cancer

Correspondence: Keiichi Tamai, Division of Cancer Stem Cell, Miyagi Cancer Center Research Institute, 47-1 Nodayama, Medeshima-Shiode, Natori, 981-1293 Japan. E-mail: tamaikeiichi@med.tohoku.ac.jp

Tel: +81-22-381-3151

Fax: +81-22-381-1168

Supplementary Figure S1

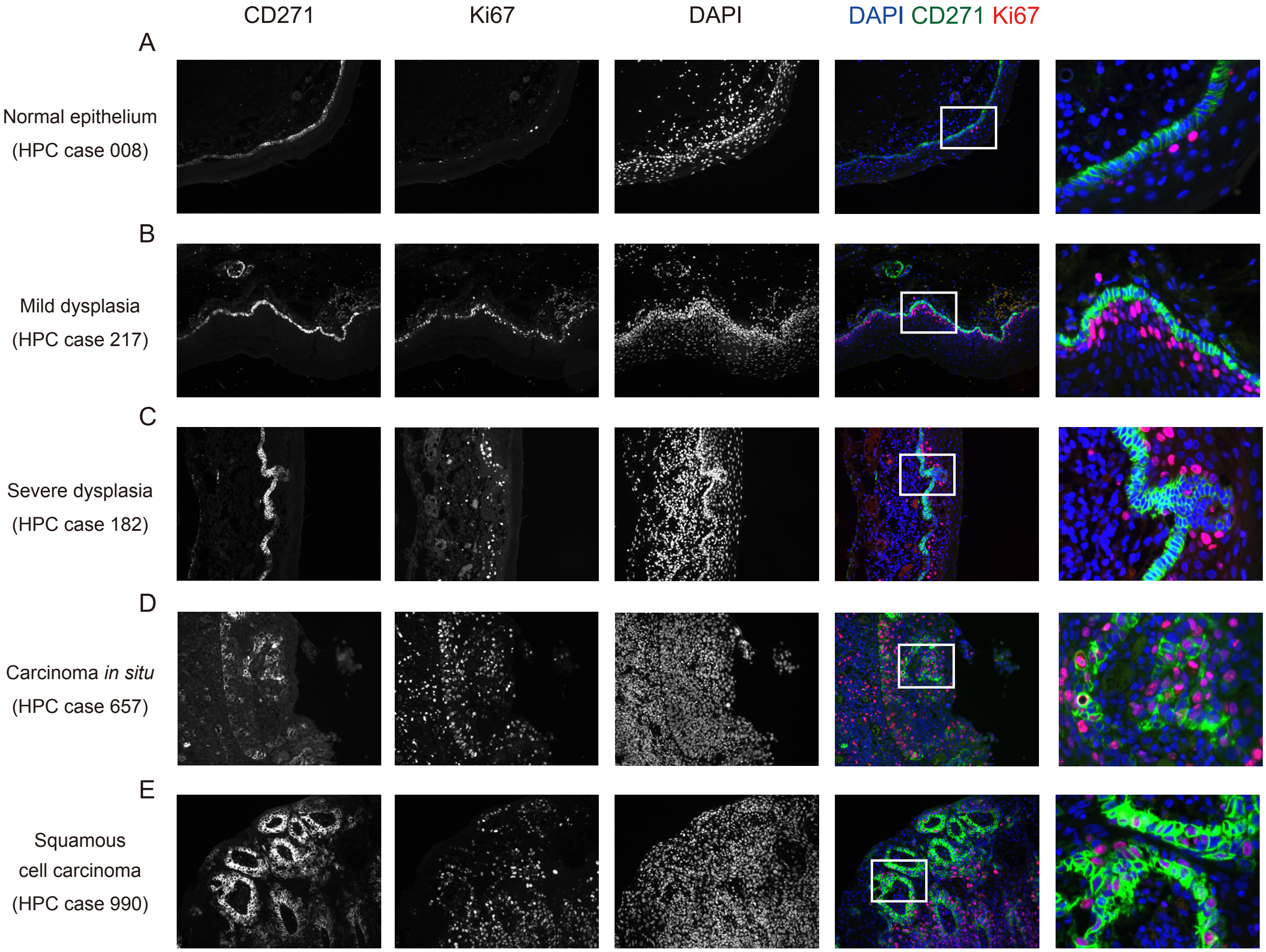

Supplementary Figure S1
Representative images of the immunofluorescence staining of HPC specimens. Immunostaining was performed with anti-CD271 and anti-Ki67 antibodies and DAPI. Far right panels are high-magnification images. The specimens were pathologically diagnosed as normal epithelium (A), mild dysplasia (B), severe dysplasia (C), carcinoma in situ (D), and squamous cell carcinoma (E).

Supplementary Figure S2

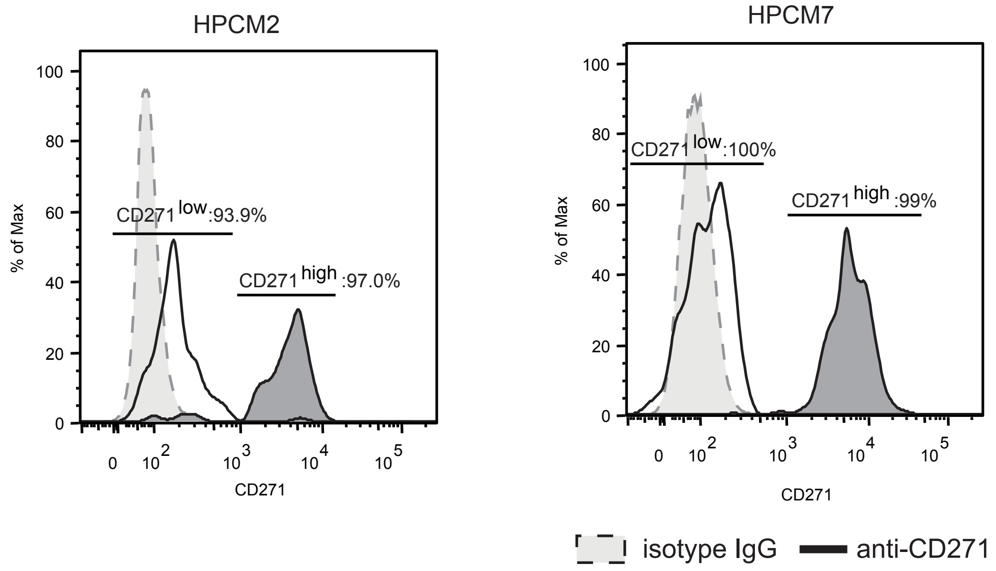

Supplementary Figure S2

HPCM2 and HPCM7 cell lines were sorted into CD271^{high} and CD271^{low} cell populations by FACS. The percentages of the CD271^{high} cell populations that were CD271^{+} and the percentages of the CD271^{low} cell populations that were CD271^{-} are shown.

Supplementary Figure S3

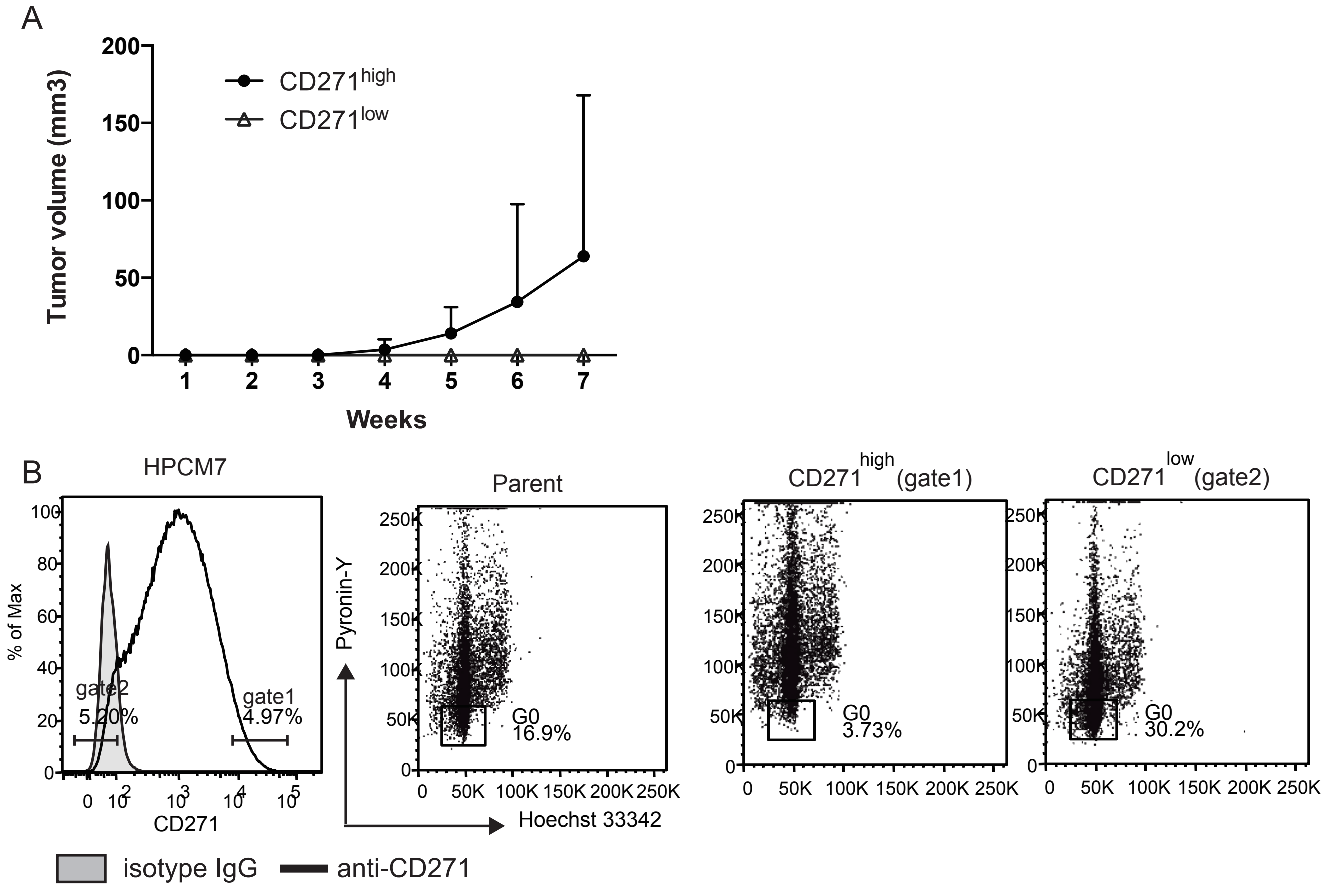

Supplementary Figure S3

A) Tumorigenic activities of the HPCM7 CD271^{high} and CD271^{low} cell populations were assessed by xenografting into NOG mice. B) Cell cycle analysis of HPCM2 cells was performed by Hoechest 33342 and pyroninY staining for 1 h at 37˚C followed by staining with an anti-CD271 antibody for 30 min at 4˚C. The gated populations of the CD271^{high} fraction (gate 1, 4.9% of the cells) and the CD271^{low} fraction (gate 2, 5.2% of the cells), and the entire population of parent HPCM7 cells were subjected to cell cycle analysis. The percentages of cells in G_0 are shown.

Supplementary Figure S4

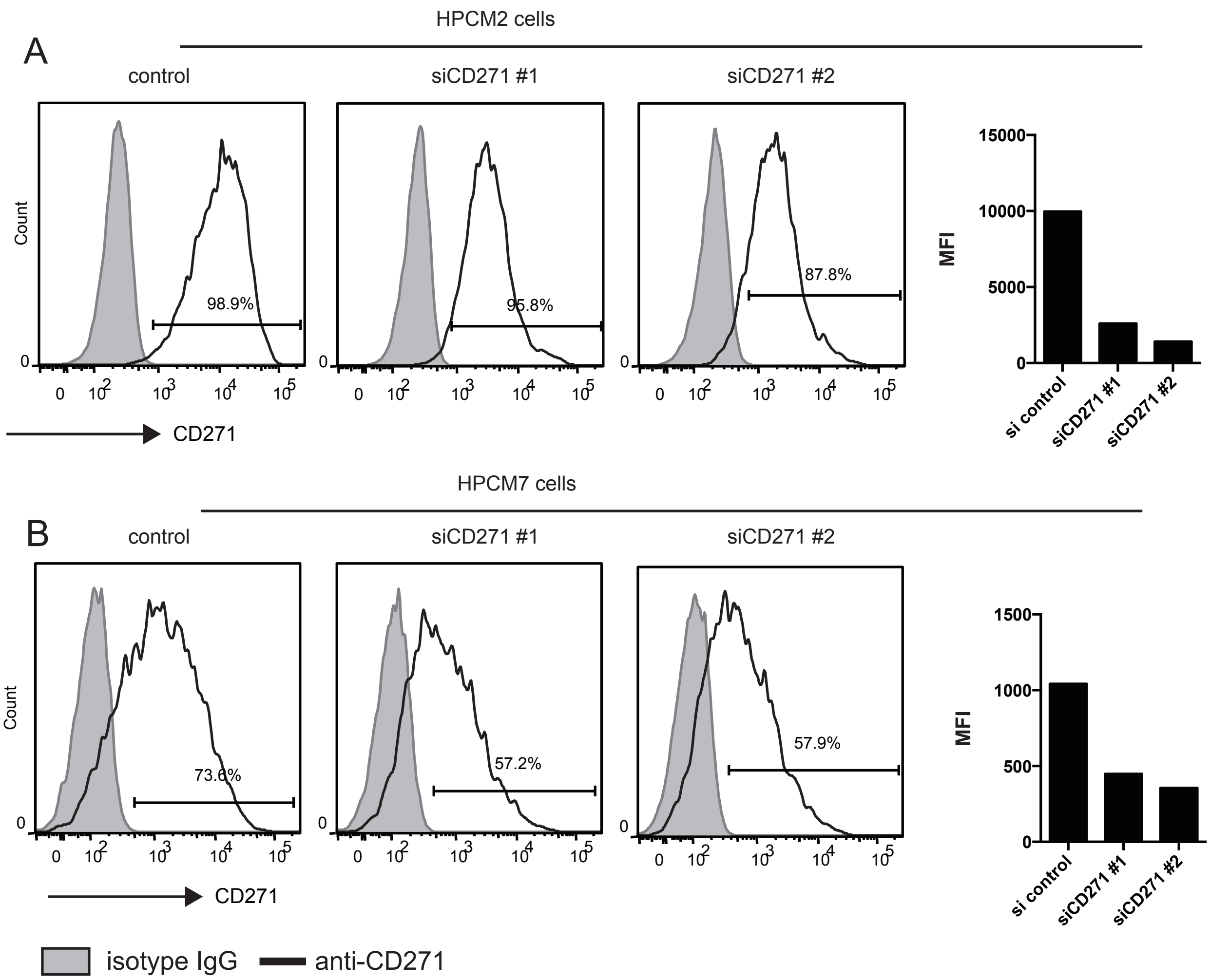

Supplementary Figure S4
Flow cytometry analysis of the CD271-knockdown cells was performed by anti-CD271 antibody staining in HPCM2 (A) and HPCM7 (B). Left panels: Representative flow cytometry histograms. The percentages of the CD271-positive cells are shown. Right panels: Median fluorescence intensities (MFI) (anti-CD271 MFI minus isotype IgG MFI).

Supplementary Figure S5

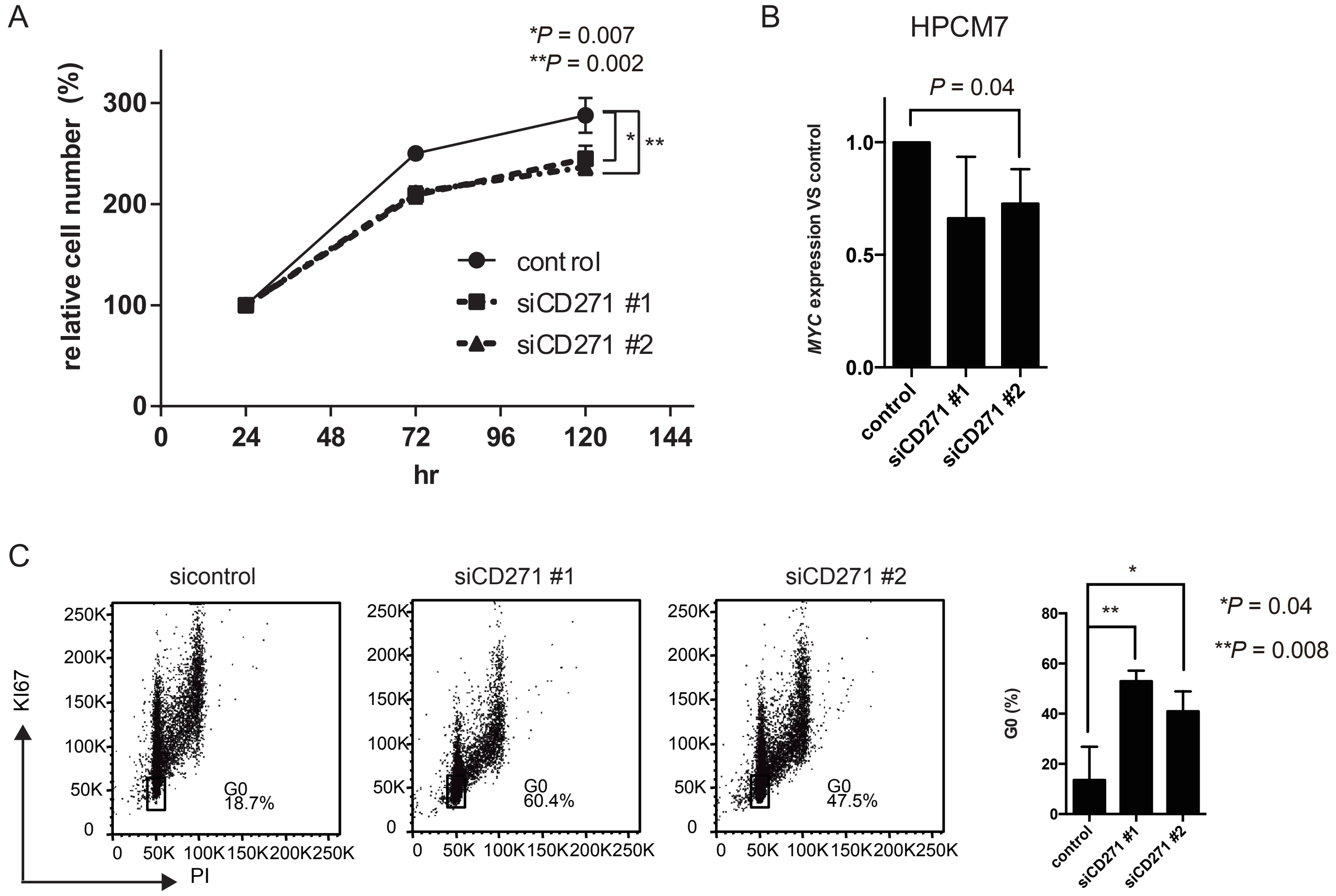

Supplementary Figure S5

A) Proliferation of CD271-knockdown (siCD271#1 and siCD271#2) and control HPCM7 cells was assessed with MTT assays. B) MYC expression in CD271-knockdown and control HPCM7 cells was analyzed by real-time PCR. C) CD271-knockdown HPCM2 cells were subjected to cell cycle analysis by staining with an anti-Ki67 antibody and propidium iodide (PI). The percentages of cells in G0 are shown in the graph (n=3).

Supplementary Figure S6

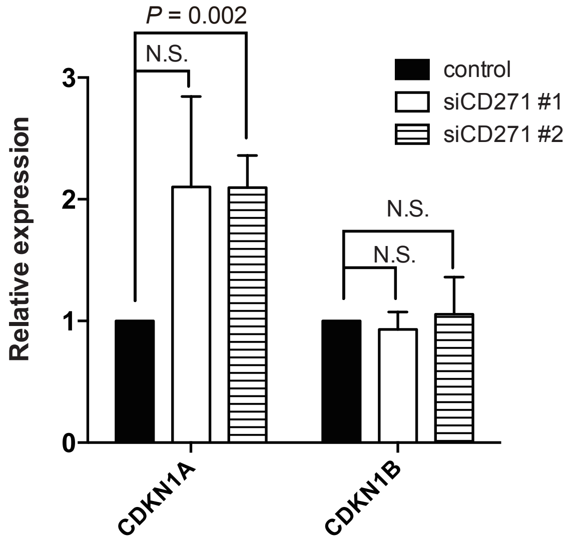

Supplementary Figure S6
Expression of CDKN1A and CDKN1B in CD271-knockdown cells and control HPCM2 cells was analyzed by real-time PCR. (n=3, N.S., not significant.)

Supplementary Figure S7

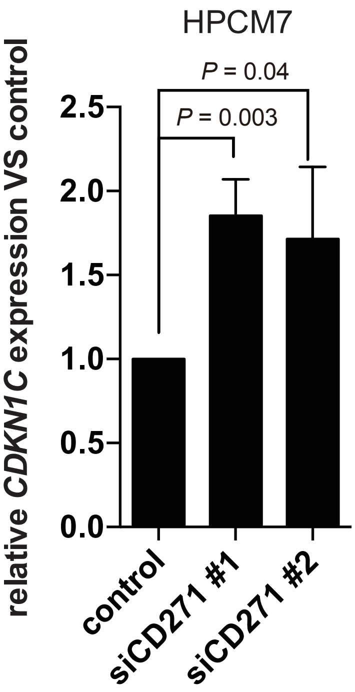

Supplementary Figure S7
Expression of CDKN1C in CD271-knockdown and control HPCM7 cells was analyzed by real-time PCR (n=3).

Supplementary Figure S8

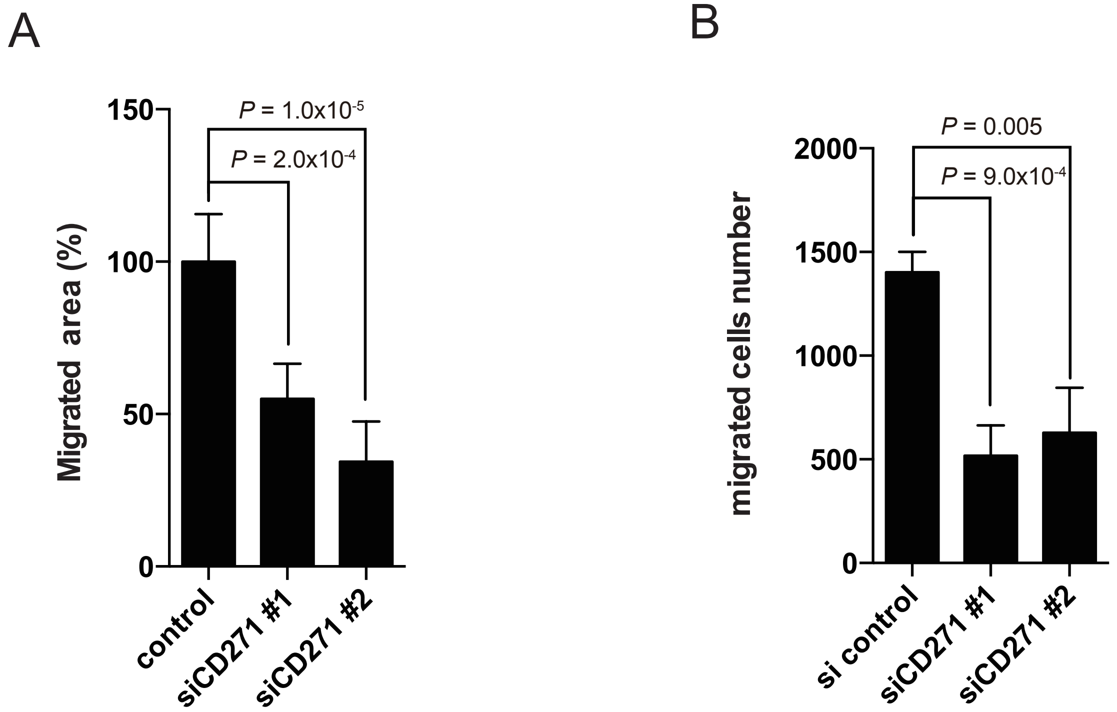

Supplementary Figure S8
A) Scratch assays. Confluent HPCM7 cells were scratched with a pipette just after transfection with siCD271#1 or #2, and the scratched areas were measured at 0 h and 14 h of culture. Quantification of the migrated areas is shown. (n=6) B) CD271-knockdown and control HPCM7 cells were incubated in a transwell unit for 24 h in medium containing 10% FBS, and then the transwell membrane was stained with Diff-Quick. Quantification of migrated cells is shown. (n=3)

Supplementary Figure S9

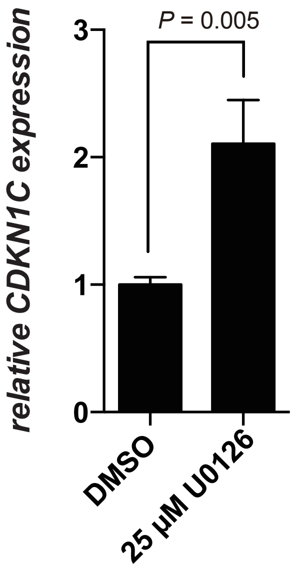

Supplementary Figure S9
CDKN1C expression in U0126-treated HPCM2 cells was quantified by real-time PCR. HPCM2 cells were incubated in complete medium containing 25 µM U0126 or vehicle (0.1% DMSO) for 72 h. (n=3)

Supplementary TableS1

Top 20 gene sets of GESA analysis.

RANK	GENE SET NAME (GO)	FDR q-val
1	DNA_REPAIR	≦ $1x10^{-4}$
2	DNA_DEPENDENT_DNA_REPLICATION	≦ $1x10^{-4}$
3	DNA_REPLICATION	≦ $1x10^{-4}$
4	DNA_METABOLIC_PROCESS	≦ $1x10^{-4}$
5	RESPONSE_TO_DNA_DAMAGE_STIMULUS	≦ $1x10^{-4}$
6	M_PHASE	≦ $1x10^{-4}$
7	NUCLEOLUS	≦ $1x10^{-4}$
8	MITOTIC_CELL_CYCLE	≦ $1x10^{-4}$
9	CHROMOSOME	≦ $1x10^{-4}$
10	CELL_CYCLE_PHASE	≦ $1x10^{-4}$
11	M_PHASE_OF_MITOTIC_CELL_CYCLE	≦ $1x10^{-4}$
12	CHROMOSOMAL_PART	≦ $1x10^{-4}$
13	SMALL_NUCLEAR_RIBONUCLEOPROTEIN_COMPLEX	≦ $1x10^{-4}$
14	REPLICATION_FORK	≦ $1x10^{-4}$
15	RNA_SPLICING	≦ $1x10^{-4}$
16	SPLICEOSOME	≦ $1x10^{-4}$
17	MITOSIS	≦ $1x10^{-4}$
18	CELL_CYCLE_GO_0007049	≦ $1x10^{-4}$
19	CELL_CYCLE_PROCESS	≦ $1x10^{-4}$
20	NUCLEOLAR_PART	≦ $1x10^{-4}$

Gene expression data were analyzed using GO gene sets.

Characters in red indicate cell cycle-related gene sets.

Supplementary TableS2

Target Gene	Forward primer	Reverse primer
β-actin	ccaaccgcgagaagatga	tccatcacgatgccagtg
CD271	tgctgttgctgcttctgg	ctcacacacggtctggttg
MYC	caccagcagggactctga	gatccagactctgaccttttgc
CDKN1A	cgaagtcagttccttgtggag	catgggyycygacggacat
CDKN1B	tttgacttgcatgaagagaagc	agctgtctctgaaagggacatt
CDKN1C	gcggcgatcaagaagctgt	atcgcccgacgacttctca

Information for primers used in this paper.
